# Overexpression of the sugar transporter gene *VvSWEET15* enhances sucrose synthase VvSUSY3-driven sucrose degradation to promote hexose accumulation in grape berries

**DOI:** 10.1093/hr/uhag206

**Published:** 2026-06-02

**Authors:** Yun Yan, Yang Xiao, Jianrong Tan, Lingcheng Zhu, Chunhua Ma, Jianhui Shao, Jing Su

**Affiliations:** College of Landscape and Horticulture, Yunnan Agricultural University, Kunming 650201, Yunnan, China; College of Landscape and Horticulture, Yunnan Agricultural University, Kunming 650201, Yunnan, China; College of Landscape and Horticulture, Yunnan Agricultural University, Kunming 650201, Yunnan, China; State Key Laboratory of Crop Stress Biology for Arid Areas, College of Horticulture, Shaanxi Key Laboratory of Apple, Northwest A&F University, Yangling 712100, Shaanxi, China; College of Landscape and Horticulture, Yunnan Agricultural University, Kunming 650201, Yunnan, China; College of Plant Protection, Yunnan Agricultural University, Kunming 650201, Yunnan, China; College of Landscape and Horticulture, Yunnan Agricultural University, Kunming 650201, Yunnan, China

## Abstract

In fruit crops, sugar accumulation is a highly orchestrated process that requires coordination among numerous sugar transporters and metabolic enzymes. However, much remains unknown about how the underlying mechanisms coordinating these genes contribute to controlling sugar movement across the apoplasmic gap and sugar accumulation in fruits. In this study, we identified a cytoplasmic membrane–localized sucrose transporter, VvSWEET15, from grapevine (*Vitis vinifera* L.), whose expression was associated with significant hexose accumulation during berry ripening and exhibited expression patterns similar to those of the sucrose synthase *VvSUSY3*. Although overexpressing *VvSWEET15* increased fructose and glucose concentrations in transgenic grape calli, it unexpectedly decreased that of sucrose. RNA sequencing (RNA-seq) and expression analyses revealed that *VvSUSY3* was up-regulated in the transgenic calli lines overexpressing *VvSWEET15*. Further analyses suggested that the increased hexose concentration in the transgenic lines correlated with the up-regulation of *VvSUSY3* expression. Suppressing *VvSUSY3* in the *VvSWEET15*-overexpressing grape calli reduced the positive effect of *VvSWEET15* on sugar accumulation. These findings demonstrate that the increased sucrose transport into the cytosol, mediated by VvSWEET15, up-regulates sucrose synthase *VvSUSY3* expression to promote hexose accumulation in the berries. Thus, our study establishes a framework for understanding how sugar transporters and metabolic genes are integrated to facilitate sugar partitioning and accumulation in plants.

## Introduction

Sugars act not only as a crucial source of carbon for plants but also as signaling molecules in response to environmental, developmental, and metabolic cues [1]. Sugars are important nutritional compounds, especially in fruit crop species, and the accumulation of soluble sugars (sucrose, fructose, and glucose) contributes to the flavor and quality of fruits [2, 3]. However, sugar transport and partitioning are required to accumulate these sugars, and specialized sugar transporters play an indispensable role in this process.

To date, three sugar transporter protein families have been identified in plants: the monosaccharide transporters (MSTs), sucrose transporters (SUTs), and sugar will eventually be exported transporters (SWEETs) [4–6]. Among them, SWEETs are a novel class of sugar transporters that move sugars bidirectionally and facilitate sugar diffusion along concentration gradients, which, in turn, affect the accumulation and distribution of sugars within the plant [7, 8] In *Arabidopsis*, phylogenetic analysis indicated that the SWEET protein family is subdivided into four clades [9]: Clade I (SWEET1–2) and Clade II (SWEET3–8), which prefer hexoses; Clade IV (SWEET16–17), which is involved in the flux of fructose across the tonoplast; and Clade III (SWEET9–15), which mainly transports sucrose. The Clade III SWEET facilitation system has evolved as a ubiquitous unloading system in fruit crops, members of which have also been detected and characterized in other plant species, including apple (*Malus domestica*) [10], pineapple (*Ananas comosus*) [11], tomato (*Solanum lycopersicum*) [12], and grape (*Vitis vinifera*) [13]. Notably, the Clade III SWEET members play essential roles in regulating sugar accumulation. For instance, overexpression (OE) of *VvSWEET10* in grape calli and tomato significantly enhances glucose, fructose, and total sugar levels [14]. In apple, Zhang *et al*. confirmed that MdSWEET9b is crucial for the sugar unloading process, accelerating the transmembrane transport of sugar and ultimately promoting sucrose and total sugar accumulation in the fruit [10].

Recent advances have revealed that the plasma membrane and tonoplast-localized SlSWEET15 also facilitate sucrose import from the apoplasm to the cytosol, then to the vacuole of storage parenchyma cells for continuous intracellular sugar allocation [12]. Notwithstanding, once sucrose is imported into fruit cells, most of the sugars stored in fruit vacuoles are glucose or fructose [15]. Although sucrose transported by SWEET15 is hypothesized to be rapidly cleaved into hexoses, direct experimental evidence remains lacking. Therefore, investigating genes that contribute to SWEET-mediated sugar accumulation in fruits is warranted. Sucrose synthase (SUSY, EC 2.4.1.13) is now widely recognized as a key enzyme that hydrolyzes sucrose to provide substrates for plant metabolism, and serves as a reliable indicator of sink strength in plants [16, 17]. In tomato, Zhang *et al*. found that knocking out the calcium-dependent protein kinase 27 (SlCDPK27) suppressed SlCDPK26-mediated phosphorylation of sucrose synthase SlSUSY3, increasing fruit glucose and fructose concentrations by up to 30% [18]. A previous transcriptomic analysis involving three grape cultivars indicated that *VvSWEET15* is coexpressed with a *SUSY* gene and that high expression of these genes correlates with higher sugar content in the berries [19]. Plants likely utilize the SWEET15-SUSY system as part of an energy-independent strategy to regulate sugar accumulation. However, it remains unclear whether SWEET15 directly regulates SUSY activity in fruit.

Grapevine (*V. vinifera* L.) is one of the most widely cultivated and economically important perennial fruit species. During ripening, the berries accumulate high concentrations of glucose and fructose (~1 M each) within the vacuoles of flesh cells [20]. Previous studies have shown that this hexose accumulation in grape berries involves the coordinated activity of sucrose-metabolizing enzymes and sucrose transporters across different cellular compartments [21]. In this study, we identified a cytoplasmic membrane-localized sucrose transporter named VvSWEET15 in grapes and established that the increased sucrose transport mediated by VvSWEET15 into the cytosol up-regulated sucrose synthase *VvSUSY3* expression, thereby promoting sucrose cleavage and contributing to hexose accumulation in the berries. The results provide insights into the regulatory mechanism of sugar allocation and accumulation in fruits through the coordination of the ‘SWEET15-SUSY3’ pathway.

## Results

### The accumulation pattern of soluble sugar during ‘Red Globe’ berry development

Soluble sugars, including sucrose, fructose, and glucose, are not only essential for fruit growth and development but also central to fruit quality [4]. Pulp tissues were sampled from ‘Red Globe’ berries at four developmental stages (40, 80, 100, and 120 DAF, denoted as S1–S4) based on changes in berry weight (Fig. 1A and B). Soluble sugar concentrations were then analyzed to study the sugar accumulation in the developing grape berries. The results indicated a significant increase in fructose and glucose concentrations between 80 and 100 days after flowering (DAF), which plateaued at 120 DAF, consistent with the total soluble solids (SSC) measurements (Fig. 1C). In contrast, sucrose concentrations increased gradually from 40 to 120 DAF, albeit at low levels (Fig. 1D). This observed pattern of hexose accumulation during grape development was in line with prior studies [20].

**Figure 1:**
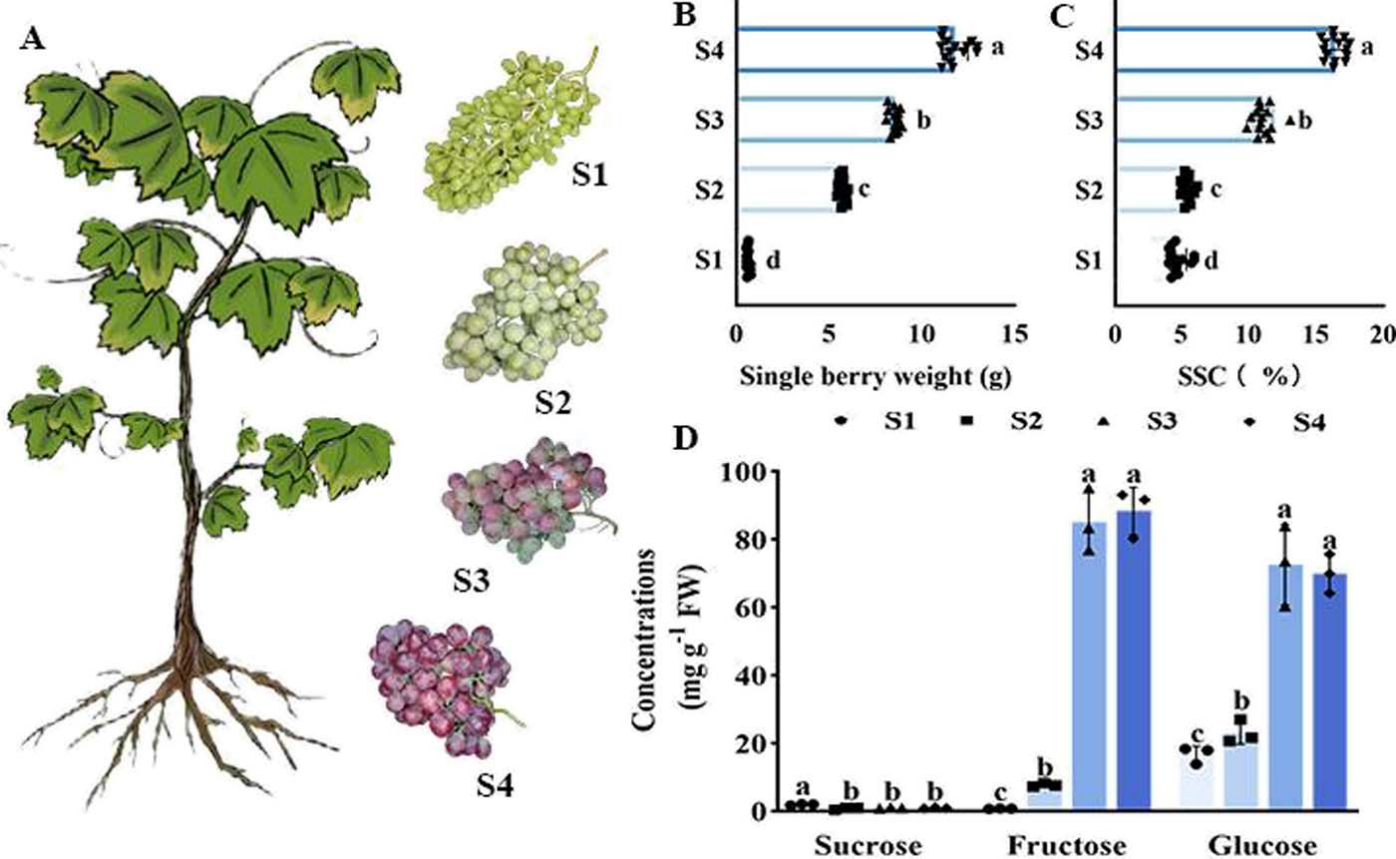
Sugar profiles in ‘Red Globe’ berries throughout their development. (A) The phenotypic changes observed in ‘Red Globe’ grapes at various developmental stages. Variations in berry weight (B) and soluble solids content (SSC) (C), respectively. (D) The concentrations of sucrose, fructose, and glucose at different phases of fruit development. Error bars represent the mean value ± standard deviation (SD) (*n* ≥ 3), with distinct letters indicating statistically significant variances at *P* < 0.05.

### Identification of potential genes involved in hexose accumulation

Transcriptome data from 40, 80, 100, and 120 DAF were analyzed to identify genes associated with hexose accumulation across all fruit developmental stages. For each sample, an average of 7.03 Gb high-quality reads were aligned to the grape genome reference (https://grapedia.org/genomes/), with an average mapping rate of 90.08% (Table S1). Principal component and correlation analyses revealed that the three biological replicates within each sample were highly similar (Fig. S1). These results confirmed the reliability and reproducibility of the RNA-seq data.

Weighted gene coexpression network analysis (WGCNA) partitioned 35 848 genes into 10 modules based on gene expression similarities (Fig. S2; Table S2). The heatmap illustrating module–trait correlations revealed that 3488 genes within the yellow and brown modules were correlated with metabolites linked to fructose and glucose (Fig. 2A). Subsequent Short Time-series Expression Miner (STEM) clustering analysis of these genes identified 20 distinct clusters (Fig. S3), with 1398 genes in clusters 12, 18, and 19 exhibiting expression patterns resembling hexose accumulation during fruit development (Fig. 2B; Table S3). Among these, pairwise comparisons S1-vs-S3, S1-vs-S4, S2-vs-S3, S2-vs-S4, and S3-vs-S4 identified a total of 272 up-regulated differentially expressed genes (DEGs) that are the ones in common (Fig. 2C, Table S4). Following Gene Ontology (GO) annotation, eight DEGs were associated with carbohydrate metabolism and transport (Fig. 2D; Table S5). Most significantly, the transcript levels of *Vitvi01g01719* (*VvSWEET15*) were significantly up-regulated from véraison through the ripening stages (Fig. 2E), thereby warranting its selection as a candidate gene for further investigation.

**Figure 2:**
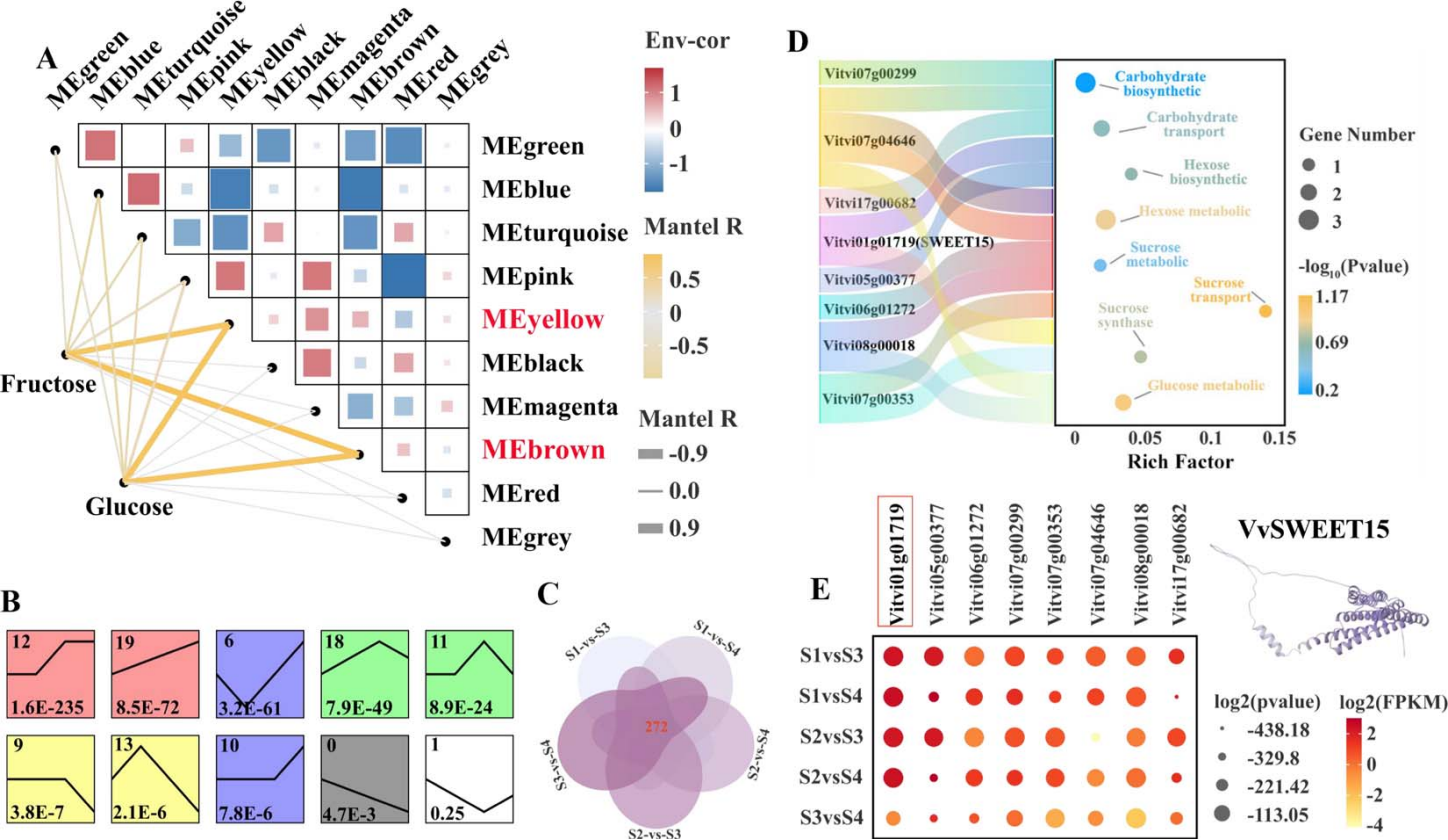
Identification of key genes for hexose accumulation in ‘Red Globe’ berries. (A) Module–trait correlation analysis using transcriptome data from ‘Red Globe’ grapes at four developmental stages, S1–S4. The width of the edges in the network diagram corresponds to Mantel’s *r* statistic, mirroring the strength of the distance correlations, with line colors indicating statistical significance. Spearman’s correlation coefficients between 10 modules and hexoses were visualized using a gradient color bar. (B) STEM clustering analysis on 3488 genes within the two modules. The gene expression levels were normalized using log2(FC), and the expression trends were categorized into 20 profiles through dynamic trend analysis, considering a significant expression trend at *P* < 0.05 and a fold change ≥1. (C) Differentially expressed genes (DEGs) identified among the groups S1-vs-S3, S1-vs-S4, S2-vs-S3, S2-vs-S4, and S3-vs-S4. (D) Gene Ontology (GO) enrichment analysis on the set of up-regulated DEGs identified from the group comparison analysis in (C). (E) Expression patterns of candidate genes related to carbohydrate metabolism and transport in ‘Red Globe’ berries across different developmental stages.

### VvSWEET15 is a cytoplasmic membrane-localized sucrose transporter protein

The amino acid sequence of Vitvi01g01719 from the ‘Red Globe’ grape variety showed high similarity to AtSWEET15 (AT5G13170) in *Arabidopsis*, which harbors seven typical transmembrane domains (TMs) and two MtN3 domains (Fig. S4). It was therefore named VvSWEET15. To further explore the role of *VvSWEET15*, its transcript level was monitored in various organs of the plants. The findings revealed that in mature fruits, *VvSWEET15* mRNA levels increased notably from 100 to 120 DAF when the sugar content was increasing (S2–S4) (Fig. 3A), indicating that *VvSWEET15* transcript levels were positively correlated with sugar concentrations (*R*^2^ = 0.85) during fruit development (Fig. 3B). These results align with prior research highlighting the predominant role of *VvSWEET15* in hexose accumulation during the crucial stages of berry development.

**Figure 3:**
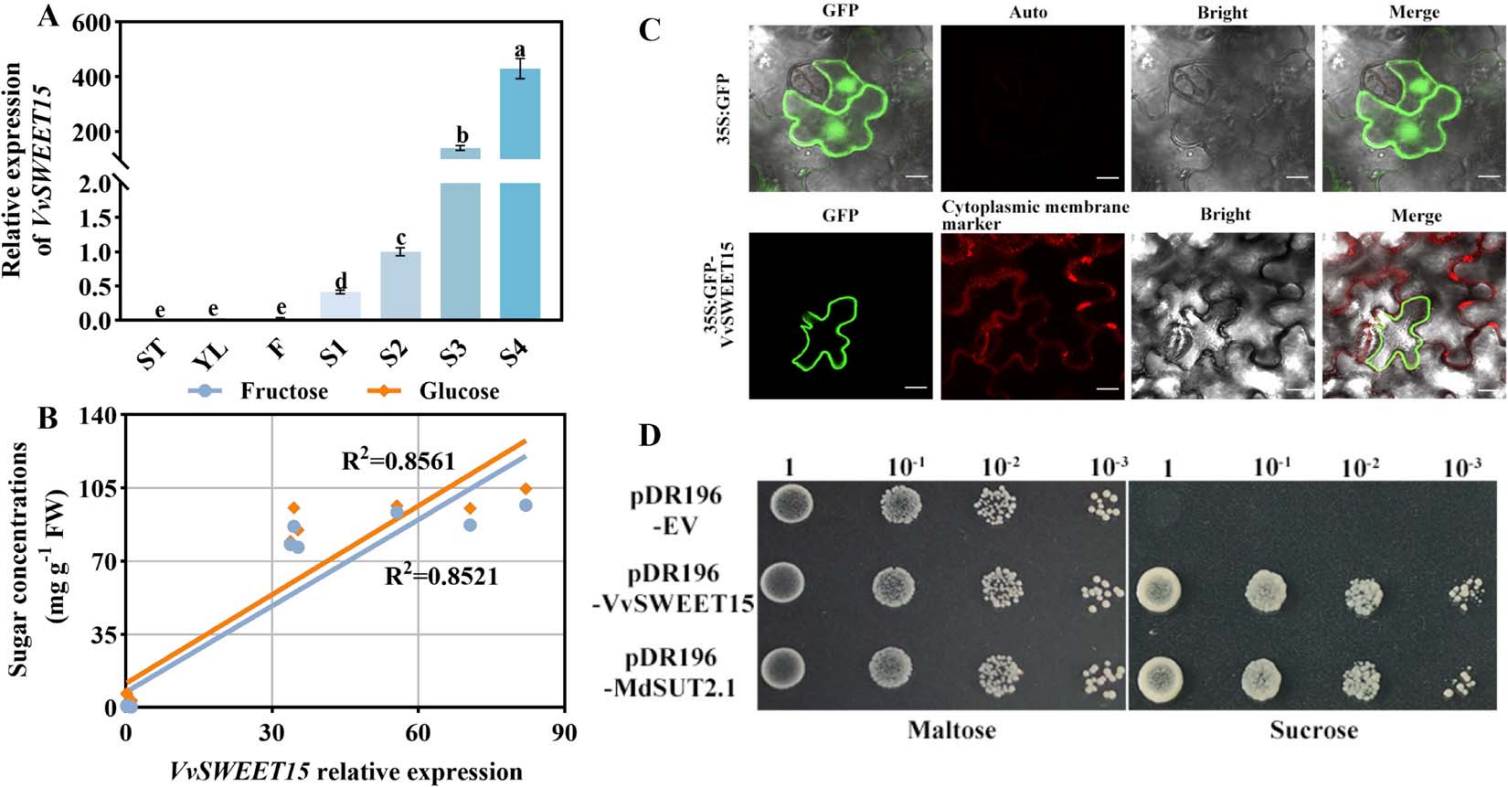
Characterization and functional analysis of VvSWEET15. (A) Relative expression of *VvSWEET15* in ‘Red Globe’ grape shoot tips (ST), young leaves (YL), flowers (F), and fruit flesh tissues at different development stages via RT-qPCR. S1: 40 days after flowering. S2: 80 days after flowering. S3: 100 days after flowering. S4: 120 days after flowering. (B) Correlation analysis between *VvSWEET15* expression levels and sugar concentrations during development. Higher R^2^ values indicate stronger correlations. FW, fresh weight. (C) Image of 35S:GFP-VvSWEET15 subcellular localization in *N. benthamiana* leaves. Scale bars indicate 20 μm. (D) Functional validation of VvSWEET15 in sucrose and hexose transporter-deficient CSY4000 yeast mutants. Yeast growth assay of CSY4000 transformed with pDR196-EV, pDR196-VvSWEET15, and pDR196-MdSUT2.1 on 2% maltose and sucrose media. Images were captured after incubation at 30°C for 4 days. Bars represent the mean value ± SD (*n* ≥ 3). Different letters indicate significant differences at *P* < 0.05.

Since AtSWEET15 belongs to the SWEET Clade III subfamily, which includes members identified as sucrose transporters localized to the plasma membrane [9], we hypothesized that VvSWEET15 is also localized to the cytoplasmic membrane. To explore this possibility, the subcellular localization of the VvSWEET15 protein was examined via laser confocal microscopy using *Nicotiana benthamiana* leaf epidermal cells. As shown in Fig. 3C, the 35S: green fluorescent protein (GFP)-VvSWEET15 fluorescence localized to the cytoplasmic membrane (Fig. 3C). Given that the amino acid sequence of VvSWEET15 and the *Arabidopsis* homolog AtSWEET15, a sucrose transporter, were highly similar, the transport activity of VvSWEET15 was assessed using the hexose and sucrose transport-deficient yeast strain CSY4000. The pDR196-empty vector (EV) and pDR196-MdSUT2.1 served as negative and positive controls, respectively. The study found that yeast cells expressing *VvSWEET15* and *MdSUT2.1* grew well on 2% (w/v) sucrose medium, compared to cells containing an EV control (Fig. 3D), while showing no growth on either 2% (w/v) fructose or glucose sugar media (Fig. S5). This growth pattern provided evidence that VvSWEET15 functioned as a cytoplasmic membrane-localized sucrose transporter.

### *VvSWEET15* promotes hexose accumulation in ‘Cabernet Sauvignon’ calli

Plants arising from stable transgenic grape tissue cultures need 8–10 years before yielding fruit. Attempts to overexpress or silence *VvSWEET15* were directly performed on ‘Red Globe’ berries to verify its roles in hexose accumulation (Fig. S6A). Transient OE of *VvSWEET15* increased their expression by 74.10%, leading to significant increases in the concentrations of fructose and glucose (Fig. S6B–D). Conversely, targeted gene suppression through the virus-induced gene silencing (VIGS) approach significantly decreased *VvSWEET15* transcript level (0.57-fold of the pTRV2 control), with the glucose and fructose concentrations reduced to 73.71% and 76.61% of the pTRV2 control conditions (Fig. S6B–D), respectively.

To further elucidate the function of *VvSWEET15* in hexose accumulation in *planta*, a recombinant construct of pC1300s*-VvSWEET15* was used for *Agrobacterium*-mediated transformation of grapevine calli. This resulted in the generation of *VvSWEET15* OE transgenic calli, with untransformed ‘Cabernet Sauvignon’ calli (WT, wild-type) serving as the control (Fig. 4A). The expression of *VvSWEET15* and sugar concentrations did not differ between WT control and corresponding EV calli (transformed with empty vector pC1300s) (Fig. S7). To analyze the sugar concentrations, three positive lines were selected via GFP screening and quantified using Reverse transcription–quantitative polymerase chain reaction (RT-qPCR; Fig. 4B). The results revealed that the high *VvSWEET15* transcript levels in the calli were associated with elevated fructose concentrations in the three transgenic lines, with levels reaching 37.09% above the WT for OE-3, 69.19% for OE-6, and 31.19% for OE-8 (Fig. 4C). The glucose concentrations in these three OE lines increased by 30.69%, 29.72%, and 40.54% (Fig. 4D), respectively, further supporting the idea that VvSWEET15 acts as a positive regulator of hexose accumulation in grape berry tissues.

**Figure 4:**
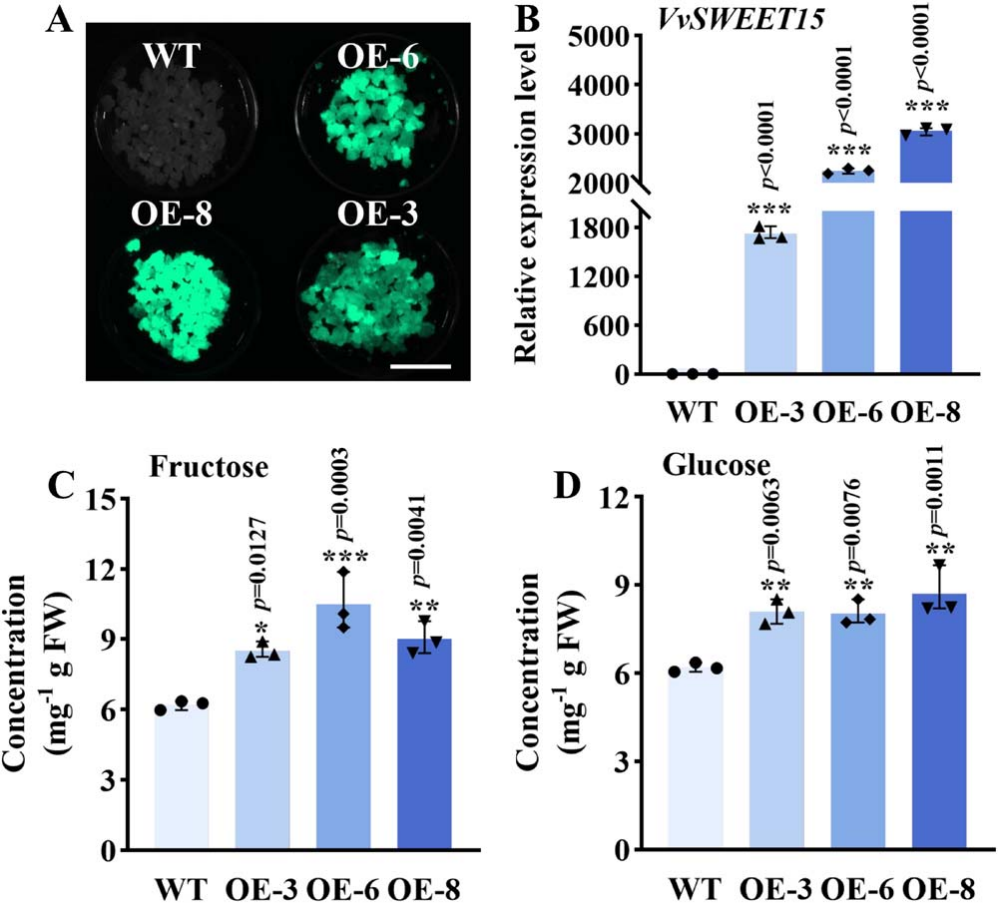
VvSWEET15 contribution to hexose accumulation. (A) Photographs of transgenic grape calli with the *VvSWEET15* overexpression (OE) and corresponding untransformed ‘Cabernet Sauvignon’ calli (WT). Scale bars represent 1 cm. (B) RT-qPCR of *VvSWEET15* expression in calli from WT and transgenic lines OE-3, OE-6, and OE-8. Concentrations of fructose (C) and glucose (D) in the transgenic lines and WT of grapevine calli. Bars represent the mean value ± SD (*n* = 3). Asterisks indicate statistically significant differences determined by Student’s *t*-test (^*^P < 0.05; ^**^P < 0.01; ^***^P < 0.001; ^****^P < 0.0001).

### *VvSUSY3* participates in *VvSWEET15*-mediated hexose accumulation

However, the sucrose concentrations of the three *VvSWEET15*-OE calli lines were significantly lower than those of the WT, with decreases of 64.34%, 79.72%, and 76.22% (Fig. 5A), respectively. One possible explanation for the observed differences is that sucrose transported by VvSWEET15 is rapidly cleaved into hexoses. To identify candidate genes in the sucrose metabolism pathway underlying the reduced sucrose levels, RNA sequencing (RNA-seq) was conducted using calli from WT and *VvSWEET15*-OE6/8. Data were filtered using log_2_(FC) > 1 relative to WT and a threshold of *P* < 0.05 to identify DEGs. In particular, the transcript level of *VvSWEET15* increased notably in both OE-6 and OE-8 compared to the WT control (Fig. 5B). Therefore, the RNA-seq results aligned with the findings from the RT-qPCR analysis.

**Figure 5:**
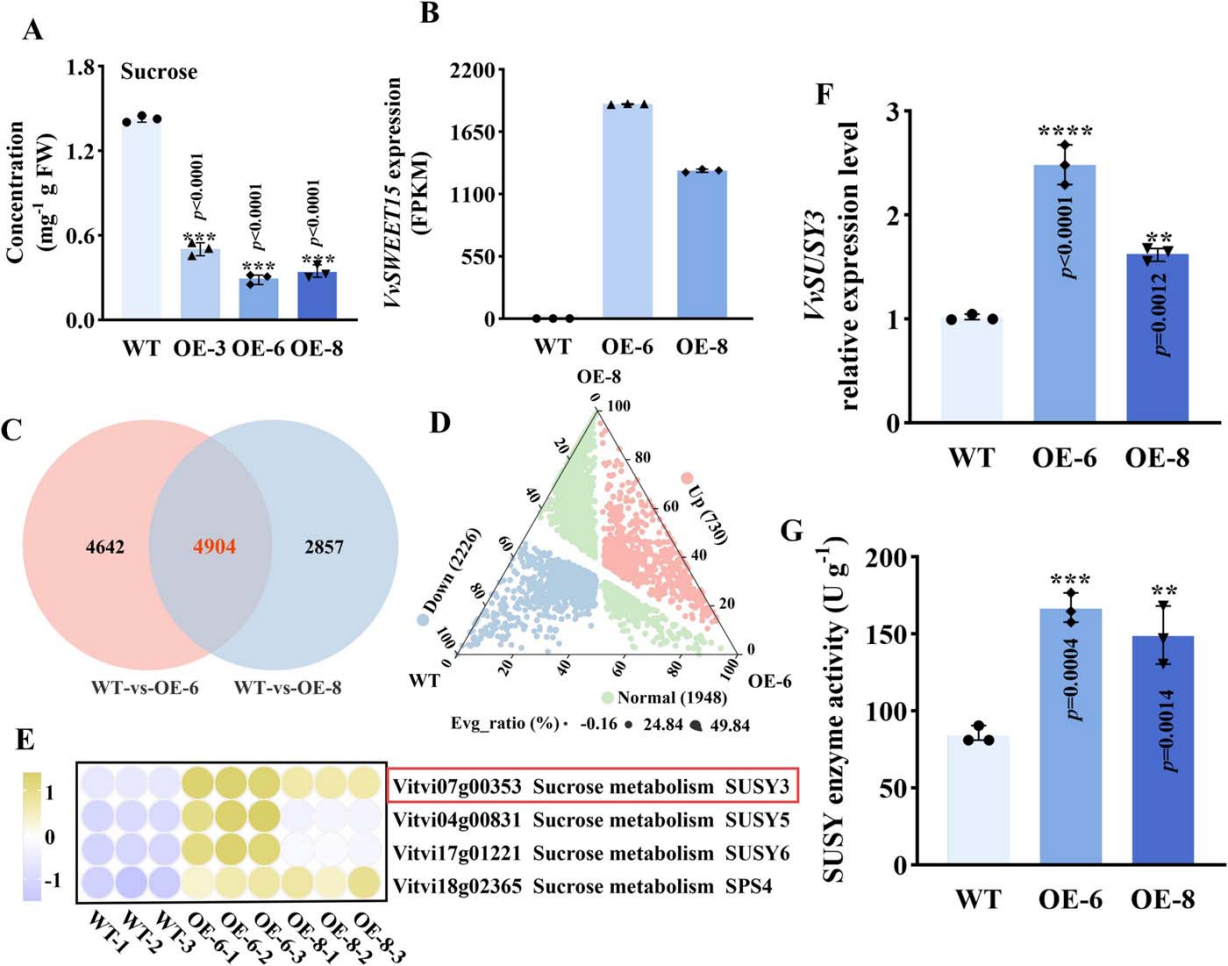
*VvSUSY3* expression patterns in the *VvSWEET15* overexpressing lines. (A) Sucrose concentration in the transgenic lines and the WT control of grape calli. (B) RNA-Seq expression profiles of *VvSWEET15*-OE and corresponding WT control calli. (C) Venn diagram analysis of common genes between different pairwise comparisons. (D) Transcriptome profiling showing up-regulated and down-regulated genes in the three overexpression lines compared to WT. (E) Heatmap showing the transcriptional abundance of four sucrose metabolism genes in *VvSWEET15*-OE and corresponding WT control calli. RT-qPCR of *VvSUSY3* expression (F) and SUSY (sucrose synthase) activity (G) in calli from WT, OE-6, and OE-8. In (F), the *VvActin* gene was used as an internal control. Bars represent the mean value ± SD (*n* = 3). Asterisks indicate statistically significant differences determined by Student’s *t*-test (^*^P < 0.05; ^**^P < 0.01; ^***^P < 0.001; ^****^P < 0.0001).

Compared with the WT, a total of 4904 genes were significantly differentially expressed in both *VvSWEET15*-overexpressing lines, of which 730 genes were significantly up-regulated and 2226 genes were significantly down-regulated (Fig. 5C and D; Table S6). Furthermore, the Kyoto Encyclopedia of Genes and Genomes (KEGG) analysis showed that four DEGs were related to sucrose metabolism (Fig. 5E), which is the process through which sucrose is degraded and resynthesized in the cytosol. Among them, sucrose synthase *SUSY3* is a key enzyme that directly hydrolyses sucrose to supply substrates for plant metabolism, which then facilitates hexose accumulation during fruit ripening [17]. Therefore, we assessed the *VvSUSY3* expression levels in the *VvSWEET15* transgenic lines using RT-qPCR, and the sucrose synthase activity of its encoding protein was confirmed in *E. coli* (Fig. S8). As depicted in Fig. 5F, the mRNA expression of *VvSUSY3* was significantly up-regulated in the three grape OE lines (Fig. 5F), leading to 1.3- to 2.0-fold increases in SUSY enzyme activity compared to WT (Fig. 5G). These results further support that *VvSUSY3* serves as a driver of fruit sugar metabolism, contributing to intracellular sugar accumulation and homeostasis.

### *VvSUSY3* expression was induced by sugar-feeding and activated by *VvSWEET15* coexpression

As shown above, the VvSWEET15-VvSUSY3 pathway might control hexose accumulation in *VvSWEET15*-overexpressing transgenic grape calli, raising the hypothesis that sucrose influx mediated by VvSWEET15 might induce the mRNA expression of *VvSUSY3*. To test this, sugar feeding with different sugar types to suspension-cultured grape calli, with the mRNA levels of *VvSUSY3* was significantly induced by feeding with 3% sucrose (Fig. S9). The findings were also supported by the positive responses of *VvSUSY3* promoter activity to exogenous sugar feeding in grape calli transiently expressing VvSUSY3P:GUS reporter gene (Fig. 6A).

**Figure 6:**
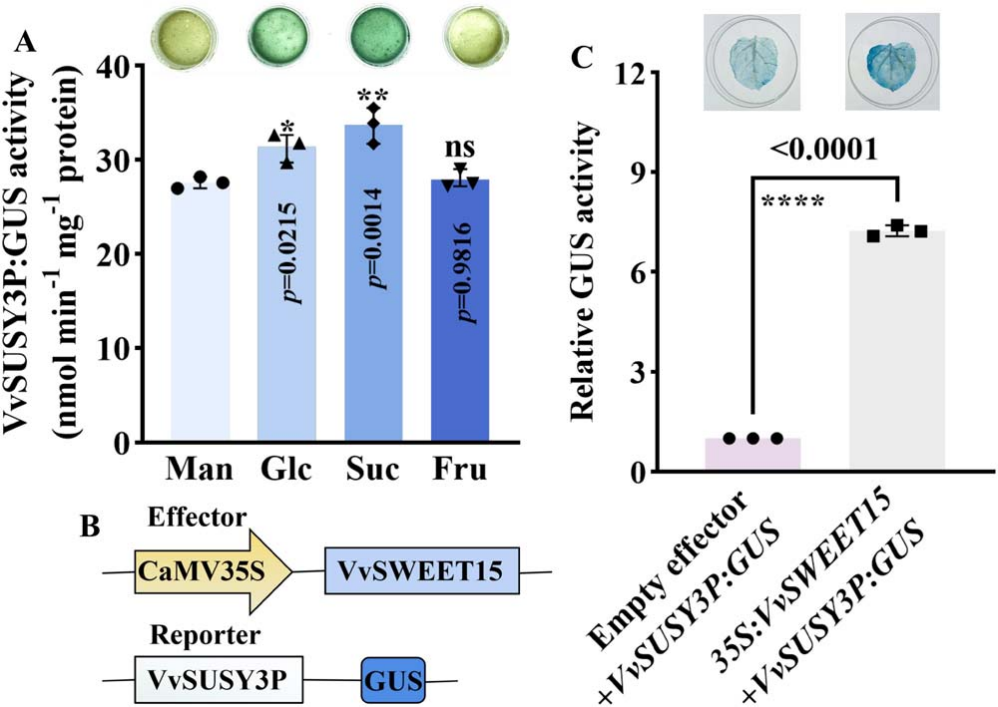
The influence of sugar feeding and coexpression of *35S:VvSWEET15* on the promoter activity of *VvSUSY3* (*VvSUSY3P*). (A) The impacts of sugar feeding on *VvSUSY3* promoter activity. Grape calli was cultured on MS liquid medium with 3% different exogenous sugars (Man, mannose; Glc, glucose; Suc, sucrose; Fru, fructose; Man was used as a control) for 24 h after infiltrating with *Agrobacterium* harbouring *VvSUSY3P*:GUS plasmids. The treated and control grape calli were harvested for GUS activity assay after 24-h feeding. (B) Schematic diagram of reporter and effector vectors for the GUS assays. (C) Impact of *35S:VvSWEET15* coexpression on *VvSUSY3* promoter activity. 35S:GUS was used as a positive control. Bars represent the mean value ± SD (*n* = 3). Asterisks indicate statistically significant differences determined by Student’s *t*-test (^*^P < 0.05; ^**^P < 0.01; ^***^P < 0.001; ^****^P < 0.0001).

To further test the influence of *VvSWEET15* expression on the transcription of *VvSUSY3*, *35S:VvSWEET15* was transiently coexpressed with *VvSUSY3P:GUS* in *N. benthamiana* leaves (Fig. 6B). The GUS staining results showed that the leaves of both the control groups (empty effector + *VvSUSY3P:GUS*) and the experimental groups (*35S:VvSWEET15* + *VvSUSY3P:GUS*) turned blue. However, the blue color of the leaves that coexpressed *35S:VvSWEET15* and *VvSUSY3P:GUS* was more intense, and its relative GUS activity was significantly higher than that of the control group (7.24-fold) (Fig. 6C), indicating that coexpression of *VvSWEET15* increases the promoter activities of *VvSUSY3*.

### Up-regulating *VvSUSY3* increased sugar concentrations in transgenic grape calli expressing *VvSWEET15*

To further assess whether the dramatically increased sugar levels in the *VvSWEET15* OE lines depend on high *VvSUSY3* expression, a VIGS approach was applied to WT and *VvSWEET15*-OE transgenic calli (Fig. 7A). The *pTRV2-VvSUSY3* construct was used to infiltrate the calli, with the empty vector pTRV2 as a control. RT-qPCR analysis revealed that the relative expression level of the *VvSUSY3* gene significantly decreased by 26.32%–36.63% in the VIGS calli compared to the empty vector (Fig. 7B), confirming that the silencing assay was successful. Orthologs of *SUSY3* have previously been confirmed as the major enzymes that directly hydrolyze sucrose to supply substrates for plant metabolism [20]. Therefore, we analyzed the SUSY activity and sugar concentration in grape calli. Infiltration of *pTRV2-VvSUSY3* in WT resulted in decreased SUSY activity and increased sucrose concentration compared with infiltration with the empty control, accompanied by a significant decrease in hexose concentration (Fig. 7C–E; Fig. S10). In the *VvSWEET15*-OE background, similar results were obtained, that is, transient interference of *VvSUSY3* reduced SUSY activity to 40.58%–42.15% and higher sucrose concentration by about half, abolishing the increase in hexose concentration in *VvSWEET15*-OE lines (Fig. 7C–E; Fig. S10). These assays indicated that the function of *VvSWEET15* in sugar accumulation in highly dependent on *VvSUSY3* in grape calli.

**Figure 7:**
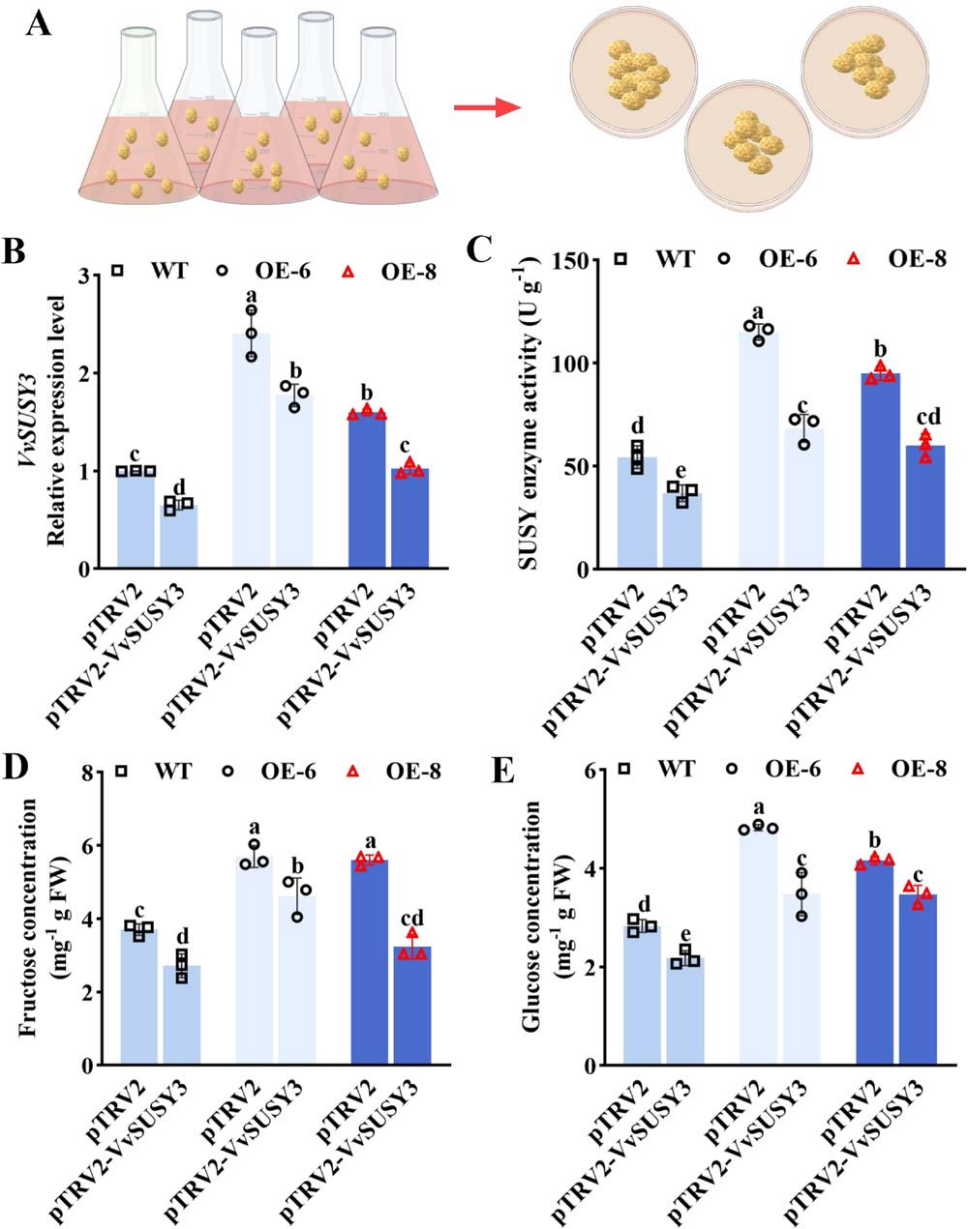
Influence of VIGS-induced *VvSUSY3* silencing on sugar levels in grape calli from either WT or *VvSWEET15* overexpression background. (A) Agroinfiltration of WT and *VvSWEET15*-OE transgenic calli. (B) RT-qPCR assay of *VvSUSY3* expression. The transcript levels were normalized to those of *VvActin*. The relative expression levels of *VvSUSY3* were obtained via the 2^−ΔΔCT^ method, setting its expression in WT infiltrated with the empty vector pTRV2 as 1. (C) SUSY enzyme activity after infiltration. (D and E) Hexose concentration after infiltration. The empty pTRV2 vector was used as a control. Bars represent the mean value ± standard deviation (*n* = 3). Different letters indicate significant differences at *P* < 0.05 based on one-way ANOVA.

**Figure 8:**
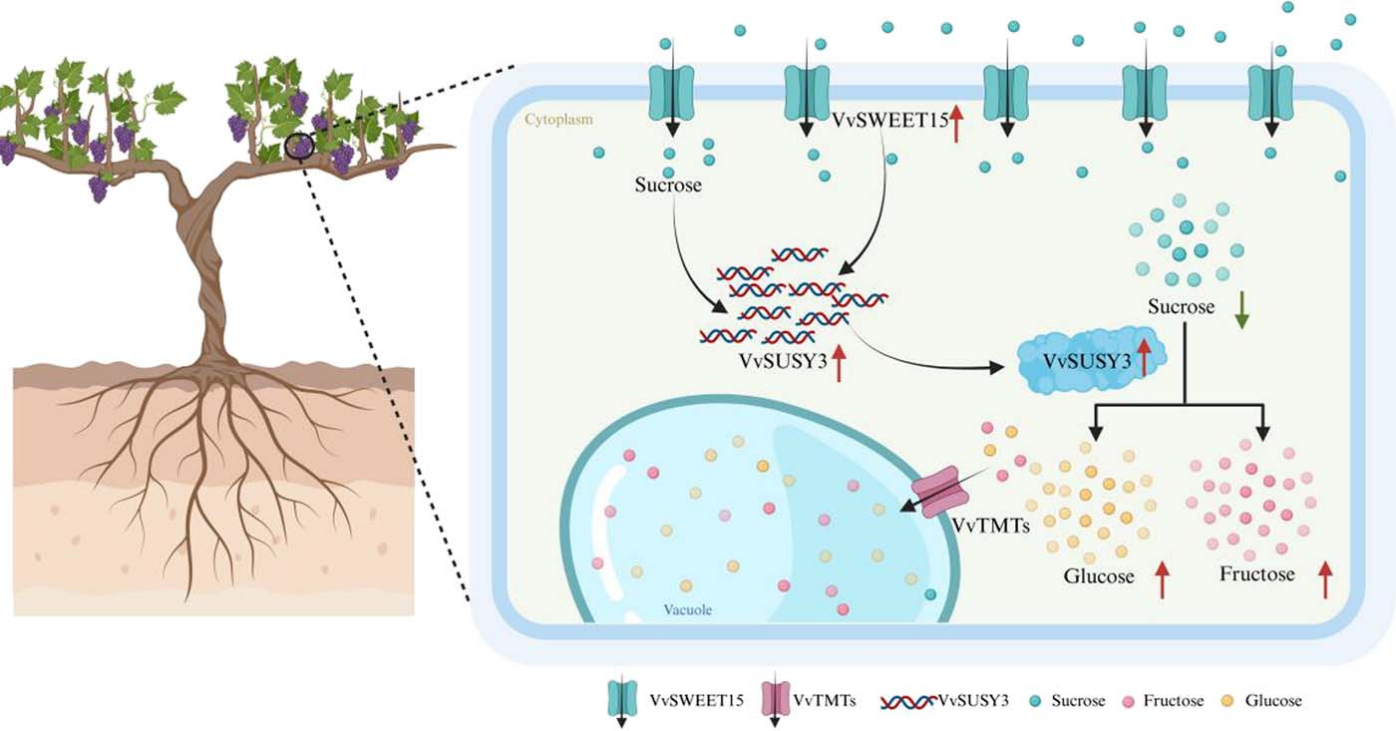
A schematic model illustrating the VvSWEET15-VvSUSY3 regulation of hexose accumulation in grape berries. Our study shows that the increased sucrose transport into the cytosol, mediated by VvSWEET15, up-regulates *VvSUSY3* expression, promoting hexose accumulation in the berries. VvTMTs, tonoplast monosaccharide transporter. Upward arrows indicate increases in sugar concentrations or relative expression levels, while the downward arrow represents a reduction in sucrose concentration.

## Discussion

### Greater contribution of hexoses to sweetness in ‘Red Globe’ grape

For a slew of fruits, such as grapes, watermelons, and apples, sugar content is a key quality indicator [22]. Grape berries are of utmost importance in the global fruit economy both as fresh fruit and as the starting material for wine production [23]. The development and ripening of the berries are commonly divided into three stages based on the different balances of sugars, acids, and phenolic compounds [24]. Among them, stage III (ripening, 100–120 DAF) is characterized by a massive accumulation of hexose in the berries. In our study, an analysis of the variations in the soluble sugar concentrations revealed that the hexose levels in ‘Red Globe’ berries surged rapidly between 80 and 100 DAF until they plateaued at 120 DAF (Fig. 1D). This trend was consistent with the very high concentrations of glucose and fructose (~1 M each) observed in the vacuoles of ripe grape berry flesh cells [25]. A prior study in strawberry (*Fragaria* × *ananassa* Duchesne) fruits also found that certain elite strawberry cultivars had particularly high concentrations of hexoses [26], suggesting that hexoses are essential for the sweetness of fleshy fruits.

### VvSWEET15 association with hexose accumulation in grape berries

However, how sugar accumulates in grape berries during fruit development is still the subject of an ongoing study. Previous studies have shown that the unloading process is the main driver of sugar accumulation in grape berries [27]. Plants can choose the most suitable strategy for unloading sucrose, with either the symplastic or apoplastic pathway being more common, depending on the structures, development stages, and species [28, 29]. Accordingly, our study showed that the ‘Red Globe’ grape variety has potential symplastic connections through plasmodesmata between the sieve element-companion cell complex (SE-CC) and surrounding parenchyma cells during the early grape berry development (40 DAF) (Fig. S11A). However, plasmodesmata were rarely observed at the ripening stage III, and when they were found, some of the plasmodesmata channels were blocked with an unknown electron-opaque material at the interface between the SE-CC complex and its adjacent flesh parenchyma cells (Fig. S11B). This suggests that an apoplasmic pathway predominates during the late stages of ripening. These findings revealed that the apoplasmic pathway is crucial for accumulating soluble sugars in the ripening fruit during stage III.

Generally, the unloading step has the advantage of facilitating the accumulation of high levels of sugars in storage cells [30]. One reason for this is that sugars can be taken up by transporters into the storage cells even against their concentration gradient. It is well known that SWEET proteins are a novel class of sugar transporters that mediate sugar accumulation in various fruits [31, 32]. Direct evidence for this phenomenon is also plentiful. For instance, VvSWEET10 has been shown to transport hexose in grapes, and overexpressing it substantially increased glucose, fructose, and the total sugar content in grape calli and tomato [14]. Previously, we also identified 16 members of the grape SWEET family and categorized them into four evolutionary clades (Clades I, II, III, and IV) [33]. In the current study, the combined results of WGCNA indicated that VvSWEET15 (Fig. 2), a member of the Clade III SWEET protein subfamily, had a strong positive correlation with the accumulation of fructose and glucose (Fig. 3B). Concomitantly, VIGS-induced suppression and overexpression of *VvSWEET15* decrease and increase the concentration of hexose within the berries, respectively (Fig. S6). This suggests that VvSWEET15 a pivotal role in hexose accumulation during berry ripening. The same approach also indicated a similar function in tomatoes. Specifically, high expression of *VvSWEET15* was shown to contribute to increased glucose and fructose accumulation in tomato fruits [13]. Similarly, our transgenic calli assay results showed that overexpressing *VvSWEET15* significantly increased the fructose and glucose concentrations in ‘Cabernet Sauvignon’ calli (Fig. 4). Therefore, multi-system functional validation through transient expression in ‘Red Globe’ berries, homologous overexpression in calli, and the heterologous expression model in tomato fruit consistently further underscored VvSWEET15’s capacity to promote hexose accumulation. In particular, the functional divergence among the SWEET15 orthologs of different species highlights evolutionary adaptations that enabled different lineages to optimize their carbohydrate partitioning strategies [34].

### Overexpression of *VvSWEET15* enhances VvSUSY3-driven sucrose degradation and promotes hexose accumulation in grape berries

An important initial finding by Ko *et al*. is that SWEET proteins also allow continuous intracellular sugar allocation by facilitating sucrose import from the apoplasm to the cytosol and, subsequently, to the vacuole of storage parenchyma cells [12]. This is supported by multiple lines of evidence in our current study. First, we discovered that VvSWEET15 is localized to the cytoplasmic membrane (Fig. 3C), which is the primary site catalyzing apoplasmic sugar transport. This observation aligns with prior studies that showed subcellular localization of SWEET15 homologs in other species, such as GhSWEET15 in cotton [35], SlSWEET15 in tomato [12], and PuSWEET15 in pear [36]. Second, as expected, we showed that VvSWEET15 transports sucrose (Fig. 3D). Third, compared to the WT, we observed that the sucrose concentration was significantly lower in *VvSWEET15*-OE calli (Fig. 5A). A possible explanation for this third observation is that overexpressing *VvSWEET15* in grapes redirects photosynthetic carbon flux from sucrose to hexose, thereby altering sugar concentrations and homeostasis in berries. Our theory is supported by prior studies that have indicated that transported sucrose is rapidly hydrolyzed into hexoses by vacuolar acid invertase or sucrose synthase. This establishes a sucrose concentration gradient between the cytoplasm and vacuole that facilitates further transport, a mechanism also observed in beet roots [37]. Following this analysis, we postulate that the mechanism connecting *VvSWEET15* to regulated hexose concentrations may be related to its ability to control sugar metabolism and carbon distribution.

Sucrose synthase (SUSY) is an important enzyme that directly hydrolyzes sucrose to supply substrates for plant metabolism [16]. Among the DEGs identified in the grapevine calli of the *VvSWEET15*-overexpressing lines, the expression of *VvSUSY3*, a homologue of *Arabidopsis AtSUSY3*, was significantly up-regulated, resulting in increased SUSY enzyme activity (Fig. 5; Fig. S8). This finding is significant because an increased rate of sucrose cleavage in the cells overexpressing *VvSWEET15* is likely to facilitate enhanced hexose accumulation in the calli. Therefore, the increased hexose and decreased sucrose levels observed in the *VvSWEET15* transgenic lines could be attributed to the up-regulated *VvSUSY3* expression and increased SUSY activity and were consistent with the following observations: (i) sucrose elicited the strongest induction of *VvSUSY3* gene expression as compared with fructose and glucose (Fig. S9). Consistently, the promoter activity of *VvSUSY3* was indeed significantly enhanced by feeding with sucrose (Fig. 6A). These findings indicate that *VvSWEET15*-mediated sucrose influx is the primary signal to activate the *VvSUSY3* expression in the cytosol, highlighting an intricate interplay between sugar signaling and transport [6, 38]. However, it remains to be determined how the cytoplasmic sucrose signals are sensed and transmitted to modulate the expression of *VvSUSY3*; (ii) concomitantly, VIGS-mediated transient silencing of *VvSUSY3* expression led to reduced fructose and glucose concentrations in *VvSWEET15-*overexpressing calli (Fig. 7D and E). This second observation is likely due to decreased sucrose cleavage in the cells overexpressing *VvSWEET15*, which was supported by the 40.58%–42.15% reduction in SUSY enzyme activity in the calli after VIGS-mediated *VvSUSY3* silencing (Fig. 7C; Fig. S10). The data obtained in this study provide further evidence for the observed decreased sucrose in the *VvSWEET15*-OE calli lines.

## Conclusions

In summary, our results suggest a working mechanism in which the increased rate of sucrose transport, mediated by VvSWEET15 into the cytosol, up-regulates sucrose synthase *VvSUSY3* expression, promoting the rate of sucrose hydrolysis and contributing to hexose accumulation in the berries (Fig. 8). These findings provide new insights into the mechanisms driving high hexose accumulation in grape berries.

## Materials and methods

### Plant materials and growth conditions

The *V. vinifera* cultivar ‘Red Globe’ used in this study was cultivated in the grapevine germplasm collection at the College of Landscape and Horticulture, Yunnan Agriculture University, in Kunming, Yunnan, China (103^。^25′N, 25^。^57′E).

Samples of ‘Red Globe’ berries were collected between 3:00 p.m. and 4:00 p.m. from three clusters per plant at four developmental stages: before veraison (40 DAF), at veraison (80 DAF), before fruit ripe period (100 DAF) and fruit ripe period (120 DAF) [39]. Within each cluster, six to eight berries were selected, with two to three berries sampled from the upper, middle, and lower sections of the cluster and combined to create a single biological replicate.

Calli from ‘Cabernet Sauvignon’ were grown on Murashige and Skoog (MS) medium containing 1.0 mg L^−1^ 6-benzylaminopurine and 1.0 mg L^−1^ 2,4-dichlorophenoxyacetic acid at 25°C in darkness, with regular subculturing every 3 weeks.

### Determination of the soluble sugar and soluble solids content

As previously outlined [40], metabolites were extracted from 100 mg of frozen samples with 75% methanol containing ribitol as an internal standard. The sample was derivatized using methoxyamine hydrochloride and *N*-methyl-*N*-trimethylsilyl-trifluoroacetamide. The resulting metabolites were then analyzed using a GC–MS-201SE system (Shimadzu, Kyoto, Japan). The soluble solids content (^°^Brix, as SSC) was measured using an Atago 0–32^°^Brix temperature-compensating refractometer (Atago Co., Tokyo, Japan).

### Protein purification and enzyme activity assays

The coding sequence of *VvSUSY3* was cloned into the pET32a vector (with a His tag). The construct VvSUSY3-His was then transformed into *E. coli* BL21 (DE3) for expression. The recombinant VvSUSY3-His protein was purified using Ni-NTA His Bind Resin (New England Biolabs). The absorbance of purified protein was measured at 595 nm. Additionally, 30 μg of purified VvSUSY3-His protein was separated by sodium dodecyl sulfate–polyacrylamide gel electrophoresis (SDS-PAGE) and stained with Coomassie Brilliant Blue (CBB) to examine the purity. The primers employed for protein purification analysis are listed in Table S7.

The SUSY activity was determined using a sucrose synthase assay kit (Decomposition Direction SS-I, Beijing Boxbio Science & Technology Co., Ltd, Beijing, China), following the manufacturer’s instructions. Briefly, the enzyme extract was incubated in a water bath (30°C for 30 min and 95°C for 10 min). Next, after the supernatants were added with 3,5-dinitrosalicylic acid buffer and reacted at 95°C for 5 min, the absorbance values of the solutions at 540 nm were measured. All experiments were conducted in triplicate, and the mean values and standard deviation were calculated from three independent biological replicates.

### Plant transformation

The vector for overexpressing *VvSWEET15* was constructed by inserting the 867-bp full-length coding sequence (CDS) of *VvSWEET15* into a pC1300s-GFP vector downstream of the CaMV35S promoter. The recombinant plasmid was transformed into the *Agrobacterium tumefaciens* strain GV3101 (pMP90), which was then used for *Agrobacterium*-mediated transformation of ‘Cabernet Sauvignon’ calli [41]. The primers used are listed in Table S7.

### RNA sequencing and weighted gene coexpression network analysis

Total RNA was extracted from frozen samples using the Tiangen polysaccharide polyphenol plant total RNA extraction kit (TIANGEN, Beijing, China), following the manufacturer’s protocol. Three biological replicates were prepared for each sample group. The concentration of extracted RNA was measured using a NanoDrop Spectrometer (ND-100 Spectrophotometer, Peqlab), and 1% agarose gel electrophoresis was carried out to assess the quality of the RNA. RNA sequencing was performed on an Illumina HiSeq 2500 platform (Gene Denovo Biotechnology Co., Guangzhou, China). Raw reads in F_ASTQ_ format were processed using F_ASTQ_ Toolkit to obtain clean reads by eliminating low-quality reads for downstream analysis. The clean reads were aligned to the *V. vinifera* PN40024.T2T (v5) reference genome using TopHat2 [42]. DEGs were identified using the DESeq2 R package with a fold change ≥1.5 and a false discovery rate < 0.05. Transcript levels were quantified using Fragments Per Kilobase of transcript per Million fragments mapped. GO enrichment analyses were performed using the OmicShare platform (https://www.omicshare.com/), with the GO terms of all annotated grapevine protein-coding genes as the background.

Grape transcripts were analyzed using the R package WGCNA to calculate their correlation coefficients with fructose and glucose [43]. The correlation coefficients of 10 modules were obtained using automatic network construction and default settings. Trait-associated hub genes of interest within modules were identified by analyzing their gene significance and module membership values. Cluster analysis was conducted using the Omicshare platform (https://www.omicshare.com/). The Short Time-series Expression Miner (STEM) method was selected to generate the clusters. Gene expression was normalized using log2(FC), and gene expression trends were partitioned into 20 profiles using the dynamic trend analysis tool, with an expression trend threshold of *P* < 0.05 and fold change ≥1.

### Reverse transcription–quantitative polymerase chain reaction assays

Total RNA was extracted from frozen grape tissues as described by Zhu *et al*. [44]. One microgram of total RNA from each sample was used to generate first-strand cDNA using Hifair® III 1st Strand cDNA Synthesis SuperMix (Yeasen, Shanghai, China). The RT-qPCR assay was performed using a previously described method [45]. The relative gene expression levels were calculated using the 2^(−ΔΔCt)^ method with the cycle threshold (Ct), with *VvActin* (GenBank: *VIT_04s0044g00580*) as the reference gene [46]. All samples were tested in three biological replicates. The primers used for RT-qPCR are listed in Table S7.

### Heterologous expression of *VvSWEET15* in yeast

To assess the sugar transport properties, the 867-bp full-length CDS of *VvSWEET15* was inserted into a pDR196 vector. The vector was then transferred into the hexose transport-deficient yeast strain EBY.VW4000 and the hexose transport and SUC2-deficient yeast strain CSY4000 via lithium acetate transformation, with the pDR196-empty vector (EV) and pDR196-MdSUT2.1 served as negative and positive controls [47, 48]. The transformed yeast cells were cultured on synthetic dropout (SD)/-Ura plates supplemented with 2% (v/v) maltose, sucrose, fructose, or glucose (Sigma-Aldrich, St. Louis, MO, USA) as the sole carbon source to test yeast growth at 30°C for 4 days. The designed primers are listed in Table S7.

### Subcellular localization of VvSWEET15

The coding sequence of *VvSWEET15* without a stop codon was cloned into a pC1300s-GFP vector downstream of a CaMV35S promoter to create the 35S:GFP-VvSWEET15 construct. 35S:GFP was included as a positive control. The 35S:GFP-VvSWEET15 and 35S:GFP plasmids were transformed into *A. tumefaciens* strain GV3101 (pMP90) and subsequently infiltrated into *N. benthamiana* leaves as previously described [49]. The GFP fluorescence of the leaves was observed within 48–60 h after infiltration using the FV1000 (Olympus, Tokyo, Japan) at a wavelength of 500–530 nm and an argon laser excitation of 488 nm (intensity 1%). All transient expression assays were repeated at least three times. The primers used in these experiments are listed in Table S7.

### GUS activity analysis

The 1947-bp promoter region derived from *VvSUSY3* was integrated into the *HindIII* and *BamHI* sites of the pC0390-GUS vector. The resulting plasmid was transformed into *Agrobacterium* strain GV3101 (pMP90). Pairwise infiltrations of the 35S:GFP-VvSWEET15 and VvSUSY3P:GUS constructs were conducted to test the GUS activity, with the VvSUSY3P:GUS construct serving as the control. Specifically, these combinations of constructs were incorporated into leaves of 3-week-old *N. benthamiana* through *Agrobacterium*-mediated infiltration and incubated for 48–72 h. For exogenous sugar feeding, the transformed and wild-type grape calli were cultured in MS liquid medium (suspension cultured) containing different types of sugar for 24 h before measurement of GUS activity and gene expression. GUS staining and activity were then quantified using a GUS enzyme activity kit (SL7161) according to the manufacturer’s instructions (Coolaber, Beijing, China). At least three biological replicates were included. The primer sequences used for vector construction are listed in Table S7.

### Transient transformation of grape berries

To overexpress or silence *VvSWEET15*, transient transformation of ‘Red Globe’ berries was performed using a previously described method [13, 40] with minor modifications. Briefly, specific *VvSWEET15* partial sequence was inserted into the pTRV2 vector, while the *VvSWEET15* coding sequence was cloned into the pC1300s-GFP vector. Agrobacterial suspensions carrying the recombinant plasmids were incubated with 200 mM acetosyringone for 3 h before injection, with all cultures adjusted to an OD_600_ of 0.6–0.8. A total of 200 μl of suspension was slowly injected at the fruit stylar apex using a 1-ml sterile syringe. Given the substantial leakage of suspension, this injection process was repeated two to three times. Each suspension containing the corresponding vectors was injected into 8–15 berry grains. Seven days after injection, the collected samples were used for gene expression and soluble sugar detection. All primers are shown in Table S7.

### Virus-induced gene silencing of *VvSUSY3* in grape calli

A 297-bp fragment of *VvSUSY3* was amplified and cloned into pTRV2 to generate pTRV2-*VvSUSY3*. The recombinant plasmids were transformed into *A. tumefaciens* strain GV3101 (pMP90) and then transiently expressed in WT and *VvSWEET15-*overexpressing calli using a previously reported method with minor modifications [50]. The grape calli were immersed in *Agrobacterium* resuspension containing the relevant fusion plasmids, subjected to vacuum infiltration for 2 min under 0.8 Mpa pressure, and the process was repeated once. The bacterial suspension on the surface of the calli was then dried with sterile paper, and the calli were cultivated on the medium. Three days after infiltration, the collected samples were used for gene expression and sugar measurement with at least three biological replicates per treatment.

### Statistical analysis

Statistical analyses were conducted using IBM SPSS Statistics 21 (SPSS, Inc., Chicago, IL, USA) and graphed using GraphPad Prism 9 (GraphPad Software, San Diego, California, USA). Data are presented as means ± standard deviation from three biological replicates. Significance levels were denoted by asterisks based on an independent *t*-test (^*^P < 0.05; ^**^P < 0.01; ^***^P < 0.001; ^****^P < 0.0001), while distinct letters indicated significant differences determined by one-way analysis of variance (ANOVA) (*P* < 0.05).

## Supplementary Material

Web_Material_uhag206

## Data Availability

The data that support the findings of this study are included in the manuscript or supplementary materials.
