# Supplementary material for: Overexpression of the sugar transporter gene VvSWEET15 enhances sucrose synthase VvSUSY3-driven sucrose degradation to promote hexose accumulation in grape berries
Source: Hortic Res. 2026 Jun 2;13(11):uhag206. doi: 10.1093/hr/uhag206 (PMC13711664; doi:10.1093/hr/uhag206)
Supplement: Supplementary file 1 [file web_material_uhag206.zip › Supplementary Figure-clean.doc]

Figure S1 Principal component analysis based on ‘Red Globe’ transcriptome data at four developmental stages.

Figure S2 Co-expression modules (clusters) identified by weighted correlation network analysis (WGCNA)

Figure S3 STEM clustering analysis on 3,488 genes within the yellow and brown modules.

Figure S4 Amino acid sequence alignment of grape VvSWEET15 and the *Arabidopsis* homologous AtSWEET15 protein.

Figure S5 Yeast growth assay of EYB4000 transformed with pDR196-empty vector and pDR196-VvSWEET15 on 2% fructose and glucose medium. Images were captured after incubation at 30°C for 4 d.

Figure S6 Transient overexpression and VIGS-induced gene silencing of *VvSWEET15* expression on sugar concentrations in ‘Red Globe’ berries. A. Phenotypes of grape berries injected with overexpression vector for *VvSWEET15* (pC1300s-*VvSWEET15*) with empty pC1300s vector as control, and silencing vector for *VvSWEET15* (pTRV2-*VvSWEET15*) with empty pTRV2 vector as control. The red arrows show the pinhole site. Scale bars, 1 cm. B-E. The relative expression of *VvSWEET15* and sugar concentrations in berries injected with *VvSWEET15* overexpression or silencing constructs. Bars represent the mean value ± SD (n=3). Different letters indicate significant differences at P < 0.05 based on one-way ANOVA.

Figure S7 Changes in *VvSWEET15* transcript levels and sugar concentrations in calli of untransformed ‘Cabernet Sauvignon’ calli (WT) and EV (transformed with empty vector pC1300s). A. Photographs of WT control and corresponding EV calli. Scale bars, 1 cm. B. The relative expression levels of *VvSWEET15* in WT control and corresponding EV calli. C-E. The concentration of fructose, glucose, and sucrose in WT control and corresponding EV calli. Bars represent the mean value ± SD (n=3). Asterisks indicate statistically significant differences determined by Student’s *t*-test (^*^*P* < 0.05; ^**^*P* < 0.01; ^***^*P* < 0.001; ^****^*P* < 0.0001).

Figure S8 Purification of VvSUSY3 protein and sucrose synthase activity analyses.

A. M, marker. P_1_, P_2_, P_3_, and P_4_, the recombinant VvSUSY3-His protein. B and C. Recombinant proteins purified from *Escherichia coli* were subjected to sucrose synthase activity assay. EV, empty vector pET32a control. Bars represent the mean value ± SD (n=3). Asterisks indicate statistically significant differences determined by Student’s *t*-test (^*^*P* < 0.05; ^**^*P* < 0.01; ^***^*P* < 0.001; ^****^*P* < 0.0001).

Figure S9 The impacts of sugars feeding on *VvSUSY3* expression levels. Grape calli was cultured on MS liquid medium with 3% different exogenous sugars (Man, mannose; Glc, glucose; Suc, sucrose; Fru, fructose; Man was used as a control) for 24 h, and the gene expression levels were detected by RT-qPCR. The transcript levels were normalized with those of *VvActin*. Relative expression level was obtained via the 2^−ΔΔCT^ method, setting the expression level in Man feeding as 1. Bars represent the mean value ± SD (n=3). Asterisks indicate statistically significant differences determined by Student’s *t*-test (^*^*P* < 0.05; ^**^*P* < 0.01; ^***^*P* < 0.001; ^****^*P* < 0.0001).

Figure S10 Influence of VIGS-induced *VvSUSY3* silencing on sucrose level in grape calli from either WT or VvSWEET15 overexpression background. The empty pTRV2 vector was used as a control. Bars represent the mean value ± SD (n=3). Different letters indicate significant differences at P < 0.05 based on one-way ANOVA.

Figure S11 The ultrastructure of phloem cells in the vascular bundle of ‘Red Globe’ berries. A. The SE/CC complex and flesh parenchyma cells (PC) surrounding the vascular bundle during the early berry development (40 DAF). Red arrows indicate a number of plasmodesmata (PD) present between PCs. B. A transverse section of the SE/CC complex at the stage III (ripening, 100-120 DAF), showing some plasmodesmata were blocked by the electron-opaque globules (indicated by yellow arrow). Bars, 2 µm. SE, sieve element. CC, companion cell.
